# (4R,6S)-2-Dihydromenisdaurilide is a Butenolide that Efficiently Inhibits Hepatitis C Virus Entry

**DOI:** 10.1038/srep29969

**Published:** 2016-07-18

**Authors:** Chueh-Yao Chung, Ching-Hsuan Liu, Guey-Horng Wang, Alagie Jassey, Chia-Lin Li, Lei Chen, Ming-Hong Yen, Chun-Ching Lin, Liang-Tzung Lin

**Affiliations:** 1 Graduate Institute of Natural Products, College of Pharmacy, Kaohsiung Medical University, Kaohsiung, Taiwan; 2 Department of Microbiology and Immunology, School of Medicine, College of Medicine, Taipei Medical University, Taipei, Taiwan; 3 Research Center of Natural Cosmeceuticals Engineering, Xiamen Medical College, Xiamen City, China; 4 Graduate Institute of Medical Sciences, College of Medicine, Taipei Medical University, Taipei, Taiwan; 5 School of Pharmacy, College of Pharmacy, Kaohsiung Medical University, Kaohsiung, Taiwan

## Abstract

Without a vaccine, hepatitis C virus (HCV) remains a significant threat, putting 170–300 million carriers worldwide at risk of cirrhosis and hepatocellular carcinoma. Although the direct-acting antivirals targeting HCV replication have revolutionized the treatment of hepatitis C, several obstacles persist, including resistance development, potential side-effects, and the prohibitive cost that limits their availability. Furthermore, treatment of HCV re-infection in liver transplantation remains a significant challenge. Developing novel antivirals that target viral entry could help expand the scope of HCV therapeutics and treatment strategies. Herein, we report (4R,6S)-2-dihydromenisdaurilide (DHMD), a natural butenolide, as an efficient inhibitor of HCV entry. Specifically, DHMD potently inhibited HCV infection at non-cytotoxic concentration. Examination on the viral life cycle demonstrated that DHMD selectively targeted the early steps of infection while leaving viral replication/translation and assembly/release unaffected. Furthermore, DHMD did not induce an antiviral interferon response. Mechanistic dissection of HCV entry revealed that DHMD could inactivate cell-free virus, abrogate viral attachment, and inhibit viral entry/fusion, with the most pronounced effect observed against the viral adsorption phase as validated using ELISA and confocal microscopy. Due to its potency, DHMD may be of value for further development as an entry inhibitor against HCV, particularly for application in transplant setting.

Hepatitis C virus (HCV) is an etiologic agent of chronic hepatitis, liver fibrosis, and end-stage liver diseases including cirrhosis and hepatocellular carcinoma. Globally, there are about 170–300 million carriers of the virus, which represents a significant medical burden. Due to the lack of an effective preventive vaccine, HCV infection is expected to cause further morbidity and mortality in the near future.

Treatment of hepatitis C has been revolutionized with the development of direct-acting antivirals (DAAs) that target HCV replication. Since the approval of the HCV protease inhibitors Boceprevir and Telaprevir in 2011, significant efforts have been made to implement the DAAs to phase out the decade-old regimen of pegylated interferon (IFN)-α (Peg-IFN-α) in combination with ribavirin (RBV) that has been sub-optimal (about 50% in response rate) against the most prevalent genotype 1 virus in the past^1^,^2^. Newer generations of DAAs include inhibitors against the HCV serine protease (ex. Simeprevir), NS5A cofactor (ex. Daclatasvir), and the viral polymerase (ex. Sofosbuvir), with various trials experimenting combination therapies with or without IFN^2^.

Despite the improvement in achieving higher rates of sustained virological response in genotype 1 patients, the application of DAAs is still currently fraught with several important obstacles including selection of resistance-associated variants and risk of potential adverse events^2^,^3^,^4^. Furthermore, the exorbitant cost of the DAAs makes these novel antivirals relatively inaccessible to the majority of the HCV-infected populations who reside in resource-poor regions^5^. In addition, the varied response against different viral genotypes and the difficult-to-treat patient groups (ex. treatment refractory, cirrhotic, human immunodeficiency virus [HIV]-coinfected, or liver transplant patients) are all issues that remain to be addressed^1^,^2^,^6^. Finally, drug-drug interaction poses another concern. For instance, acid-suppression therapies such as H_2_-receptor antagonist famotidine and the proton pump inhibitor omeprazole can decrease the concentration of the NS5A inhibitor Ledipasvir^7^. In addition, certain HIV-1 antiretroviral agents, including Rilpivirine and Efavirenz, could lead to potential adverse drug reactions when used with a triple DAA (Paritaprevir/Ritonavir, Ombitasvir, and Dasabuvir) regimen in HCV/HIV patients coadministration^8^. Given these challenges, it is therefore necessary to continuously develop novel antivirals against HCV, especially with other modes of action, to broaden the scope of treatment strategies against hepatitis C.

HCV is an enveloped single-stranded RNA member of the *Flaviviridae* family. The virus engages with various cell membrane proteins including glycosaminoglycans (GAGs), cluster of differentiation 81 (CD81), low density lipoprotein receptor (LDLR), scavenger receptor class B type I (SR-BI), claudin-1 (CLDN1), occludin (OCLN), epidermal growth factor receptor (EGFR), and Niemann-Pick C1-Like 1 (NPC1L1) to gain entry into the hepatocyte via clathrin-mediated endocytosis^9^. Once the 9.6 kb HCV genome is released into the cytoplasm by fusion of the viral and endosomal membranes, a single polyprotein is generated and subsequently processed by host and viral proteases to produce capsid, E1 and E2 glycoproteins, viroporin p7, and the non-structural proteins NS2, NS3, NS4A, NS4B, NS5A, and NS5B. Following replication, the progeny virions are assembled on lipid droplets and egress via the cholesterol synthesis pathway^10^.

Natural products have long served as an important source of antiviral discovery, including against HCV^11^. These include extracts and secondary metabolites of the *Phyllanthus* species (an indigenous herb of Southeast Asia), which have been shown to exert inhibitory effects against hepatitis B virus (HBV), herpes simplex virus (HSV), and HIV^12^,^13^,^14^,^15^,^16^,^17^. In search of novel anti-HCV agents, we previously performed an activity-based and fraction-guided drug screening analysis of *Phyllanthus urinaria* (*P. urinaria*) as a potential source of inhibitory agents against HCV infection^18^. In this study, we further examined the molecular constituents from one of the acetone extract subfractions of *P. urinaria* that showed remarkable anti-HCV activity, and evaluated their antiviral capacity against the HCV life cycle. We describe herein the isolated (4R,6S)-2-dihydromenisdaurilide, a butenolide as a potent inhibitor against HCV entry.

## Results

### (4R,6S)-2-dihydromenisdaurilide (DHMD) exhibits antiviral effect against HCV infection at non-cytotoxic concentration

**Figure 1:**
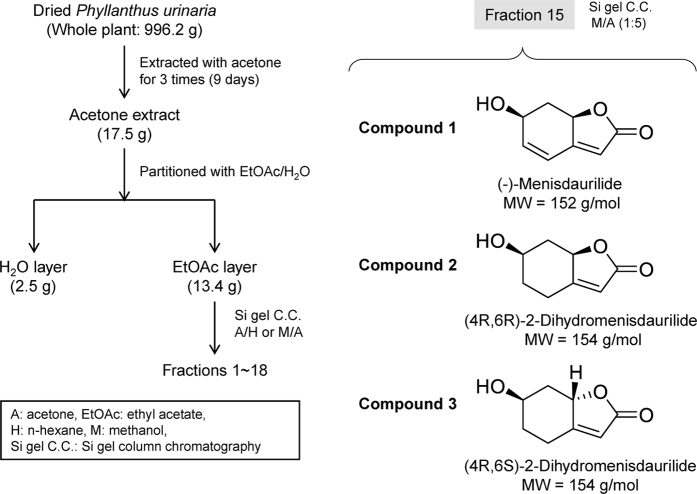
Isolation of compounds 1-3 from *Phyllanthus urinaria*. Compound **1**: (−)-menisdaurilide; compound **2**: (4R,6R)-2-dihydromenisdaurilide; compound **3**: (4R,6S)-2-dihydromenisdaurilide (DHMD).

**Figure 2:**
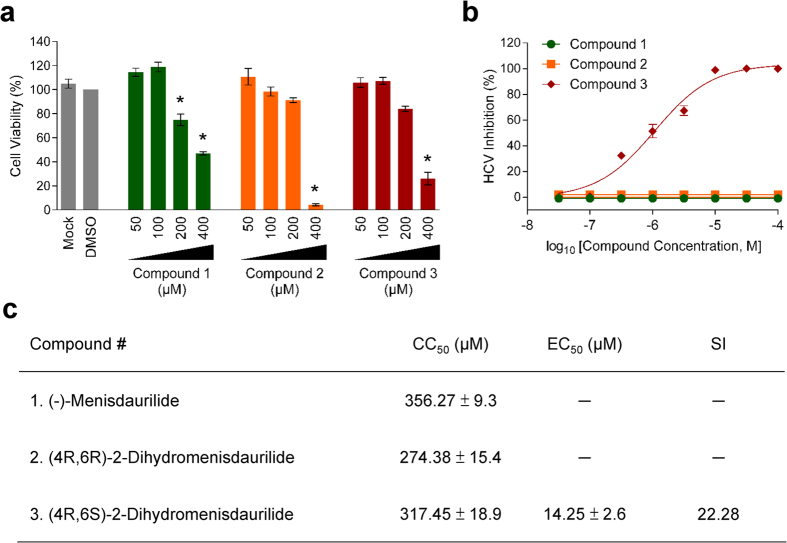
DHMD inhibits HCV infection. (**a**) Cytotoxicity (CC_50_) of the test compounds at various concentrations was tested on Huh-7.5 cell monolayers and analyzed by XTT cell viability assay. (**b**) HCV inhibition assay. Huh-7.5 cell monolayers were challenged with HCVcc (100 FFU/well) in the presence or absence of compounds **1-3** (2, 5, 10, 20, 40, 60, 80, 100 μM) and then incubated for 3 days before quantitating viral foci by immunostaining for HCV NS5A. Data are plotted against the DMSO (0.5%) negative control treatment and expressed as percent (%) HCV inhibition. Selectivity index (SI) = CC_50_/EC_50_. Data shown are means ± SEM (*P < 0.05) from 3 independent experiments.

We have previously demonstrated that several subfractions of the acetone extract of *P. urinaria* possess potent anti-HCV activity in a screening analysis^18^. As an attempt to characterize novel anti-HCV agents, one such fraction (fraction 15) was further examined in this study for its bioactive components and the associated antiviral capacity against HCV infection. Three small molecules were successfully isolated (Fig. 1) and identified as butenolides, as confirmed by their chemical structures (Supplementary Tables S1 and 2 and Figs S1–3): (−)-menisdaurilide (**1**), (4R,6R)-2-dihydromenisdaurilide (**2**), and (4R,6S)-2-dihydromenisdaurilide (**3**). Cytotoxicity analysis by XTT assay determined their half-maximal cytotoxic concentration (CC_50_) on Huh-7.5 cells as 356.27 ± 9.3, 274.38 ± 15.4, and 317.45 ± 18.9 μM, respectively (Fig. 2a). To test the compounds’ anti-HCV activity, increasing concentrations of these small molecules were mixed with HCVcc and then used to infect Huh-7.5 cells. Virus-positive foci was then analyzed and quantitated by staining for HCV NS5A. As shown in Fig. 2b, only compound **3** or (4R,6S)-2-dihydromenisdaurilide (DHMD) could significantly suppress HCV foci formation and inhibited the HCVcc infectivity in a dose-dependent manner. No activity was observed from compound **1** and **2**. The half-maximal effective concentration (EC_50_) of DHMD was determined as 14.25 ± 2.6 μM and its selectivity index (SI) against HCV was 22.28 (Fig. 2c). For all subsequent experiments, a concentration of 50 μM of DHMD was used.

### Window of antiviral activity from DHMD treatment against HCV infection

**Figure 3:**
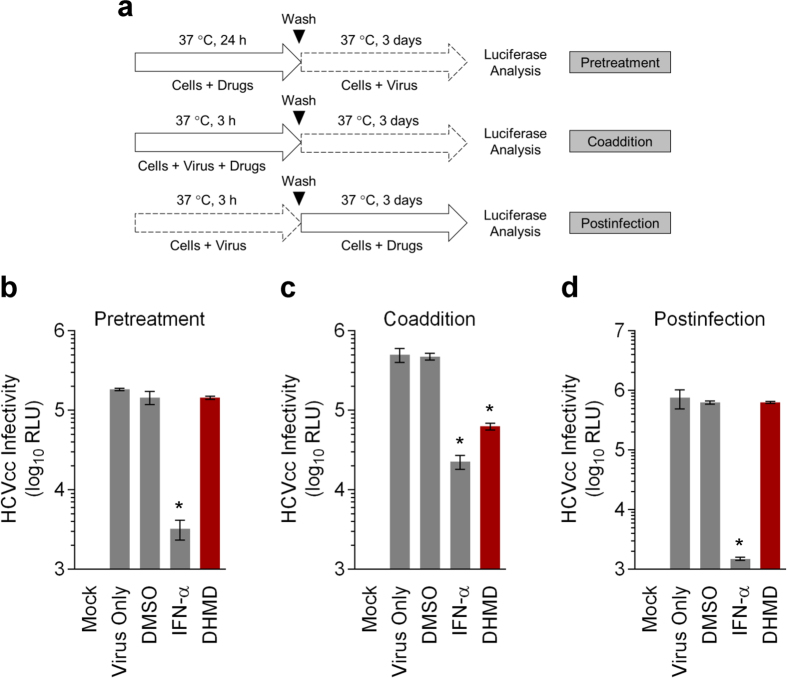
DHMD targets the early phase of HCV infection. (**a**) Schematics of the treatment analyses in the time-of-drug-addition assay. (**b**) Pretreatment effect of DHMD on Huh-7.5 cells before HCVcc infection (MOI = 0.01). (**c**) Coaddition treatment effect of DHMD and HCVcc inoculum on Huh-7.5 cells (MOI = 0.01). (**d**) Postinfection treatment effect of DHMD of Huh-7.5 cells infected with HCVcc (MOI = 0.01). Results were obtained using luciferase analysis following 72 h of incubation. DHMD = 50 μM; DMSO = 0.5%; IFN-α = 1,000 IU/ml. Data shown are means ± SEM (*P < 0.05) from 3 independent experiments.

To determine the window of antiviral activity from DHMD treatment, we next performed a time-of-drug-addition assay by adding the natural agent before, during, or after HCVcc inoculation of Huh-7.5 cells (Fig. 3a). Since the HCVcc is luciferase reporter-tagged, the measured luciferase signal following incubation correlates with HCV replication/translation upon successful infection by the virus. The results indicated that pretreatment with (Fig. 3b) or adding DHMD in the postinfection stage (Fig. 3d) had no impact on the HCVcc infection. In contrast, DHMD exhibited robust antiviral activity when it is concurrently present with the viral particles during the initial infection stage (Fig. 3c). IFN-α, which served as a positive control, successfully reduced viral reporter signals during all treatment stages.

### DHMD treatment blocks HCV early viral entry by inactivating free virus particles and inhibiting viral attachment and entry/fusion steps

The time-of-drug-addition assay suggested that DHMD might potentially mediate its antiviral activity by interfering with HCV early viral entry. To further clarify the antiviral mechanism of DHMD, its effect on the specific stages of early viral entry was examined, using a synchronized infection analysis.

**Figure 4:**
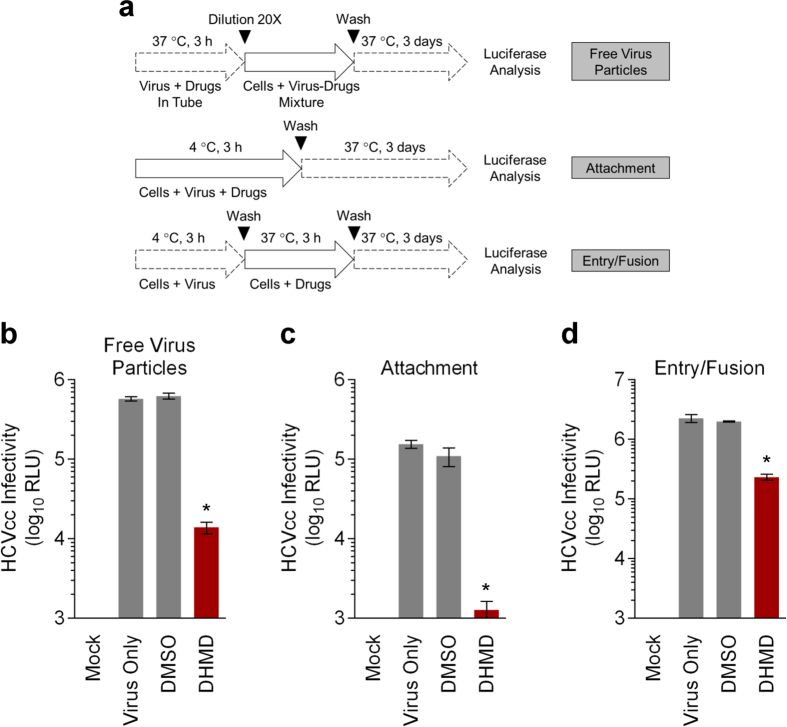
DHMD blocks HCV early viral entry by inactivating viral particles and inhibiting viral attachment and entry/fusion. (**a**) Schematics of the synchronized infection analysis on early viral entry. (**b**) Effect of DHMD on HCV free virus particles. In a cell-free condition, DHMD was mixed with the HCVcc inoculum and incubated for 3 h at 37 °C before diluting the virus-drug mixture 20-fold to ineffective concentration of DHMD for infection on Huh-7.5 cells (final viral concentration: MOI = 0.01). (**c**) Effect of DHMD on HCV attachment. Huh-7.5 cells were treated with DHMD and HCVcc (MOI = 0.01) at 4 °C for 3 h, then washed with PBS and supplemented with 2% FBS in DMEM before transferring to 37 °C for further incubation. (**d**) Effect of DHMD on HCV entry/fusion. Huh-7.5 cells were incubated with HCVcc (MOI = 0.01) at 4 °C for 3 h to allow virus adsorption, then washed with PBS and treated with DHMD at 37 °C for another 3 h before washing and refreshing the media for further incubation. All the data were obtained by luciferase analysis at the end-point (72 h) of the experiments. DHMD = 50 μM; DMSO = 0.5%; IFN-α = 1,000 IU/ml. Data shown are means ± SEM (*P < 0.05) from 3 independent experiments.

We first analyzed whether DHMD can inactivate free HCV particles prior to contact with the host cell. Cell-free virus was incubated with DHMD and then the mixture was diluted 20-fold with media to ineffective concentration of the test compound (Fig. 4a). This dilution prevents any meaningful interaction of DHMD with the target cells^19^. The virus-drug mixture was subsequently used to challenge Huh-7.5 cell monolayers before washing and determining the luciferase reporter signals following 3 days of incubation. As shown in Fig. 4b, DHMD diminished the infectivity of the virus particles, yielding reduced reporter signals.

To understand whether DHMD exhibits any impact on the viral attachment stage, we co-treated the Huh-7.5 cells at 4 °C with DHMD and the HCV inoculum (Fig. 4a). At 4 °C, the viral particles can bind to the cell surface but are precluded from entry, which occurs most efficiently at 37 °C. Treatment during viral challenge of the host cell at 4 °C therefore allows examination of the compound’s effect specifically on the viral attachment phase^19^. Following washes to remove the unadsorbed virus, the cells were incubated at 37 °C to allow the subsequent infection steps to occur and luciferase signals were determined 72 h after. Results demonstrated that DHMD robustly abrogated HCV attachment, resulting in a substantial decrease in the luciferase reporter signals of nearly 2-log difference (Fig. 4c).

To further clarify the impact of DHMD on post-binding events during early viral entry including viral entry/fusion, HCV particles were allowed to pre-bind onto the target cell surface at 4 °C. DHMD was then added to the virus-bound cells, and the temperature was shifted to 37 °C to maximize viral entry/fusion (Fig. 4a). Luciferase signals were determined following washes and further incubation at 37 °C. As depicted in Fig. 4d, DHMD also acted on the viral entry/fusion step post viral binding, albeit to a lesser magnitude compared to its effect in inactivating free virus particles and antagonizing viral attachment.

These results therefore suggest that DHMD can efficiently target several steps during HCV early viral entry, including targeting the free virus particles, blocking viral attachment, and impeding the post-binding entry/fusion step.

### DHMD treatment does not affect other stages of the HCV life cycle nor induce an antiviral immune response

**Figure 5:**
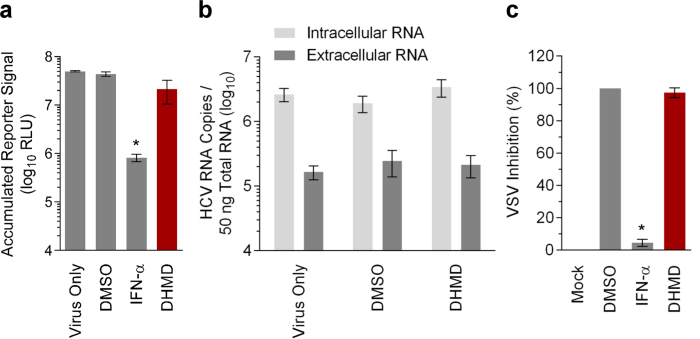
DHMD does not influence HCV replication/translation or virion production, nor induce an antiviral immune response. (**a**) Effect of DHMD on HCV replication/translation. Huh-7.5 cells were electroporated with the reporter HCV genomes and then treated with DHMD for 6 days before analyzing the supernatant for accumulated luciferase reporter signals. (**b**) Effect of DHMD on HCV assembly/release. Huh-7.5 cells established with HCV genomes were treated with DHMD for 6 days before extracting intracellular and extracellular RNAs from the cells and supernatants, respectively, and quantitated for HCV genome by qRT-PCR. (**c**) Effect of DHMD in inducing antiviral immune response against the VSV infection. Huh-7.5 cells were pre-incubated with DHMD at 37 °C for 24 h before infection with VSV-EGFP (200 PFU/well). Fluorescent viral plaques were quantitated 24 h postinfection and data are expressed as percent (%) VSV inhibition. DHMD = 50 μM; DMSO = 0.5%; IFN-α = 1,000 IU/ml. Data shown are means ± SEM (*P < 0.05) from 3 independent experiments.

To evaluate whether DHMD possesses additional antiviral activity against other stages of the HCV life cycle, we first examined its effect on viral replication/translation. For this purpose, Huh-7.5 cells were transiently established with HCV reporter genome and then treated with DHMD before assessing the accumulated luciferase signals at the end-point of incubation. The generated luciferase reporter correlates with viral genome replication/translation^20^. As shown in Fig. 5a, treatment with DHMD had little impact on the luciferase reporter signal whereas IFN-α effectively decreased its level.

To investigate the effect of DHMD on virion production, Huh-7.5 cells established with the HCV genome were treated with DHMD and then harvested for the quantitation of intracellular and extracellular HCV RNA by quantitative reverse transcription polymerase chain reaction (qRT-PCR). The extracellular RNA would correspond to the released progeny virions^21^ and the intracellular RNA level was included for comparison. Again, results showed that DHMD did not suppress HCV virion production as levels of the intracellular or extracellular viral RNA remained unchanged with or without the compound treatment (Fig. 5b).

Finally, the ability of DHMD to induce an antiviral innate immune response was evaluated using the IFN sensitive vesicular stomatitis virus (VSV)-based plaque reduction assay^22^,^23^. Huh-7.5 cells were pretreated for 24 h with the test samples, then washed and infected with enhanced green fluorescent protein (EGFP)-tagged VSV (VSV-EGFP). As shown in Fig. 5c, no difference was observed in VSV-induced plaque formation between DHMD and the control DMSO treatment. In contrast, IFN-α effectively blocked VSV plaque formation.

Altogether, the above results suggest that DHMD does not affect other stages of the HCV life cycle (Table 1), nor does it induce an antiviral innate immune response. These observations are also consistent with those obtained from the time-of-drug-addition assay, whereby DHMD treatment had no impact against HCV infection in pre- and postinfection treatments (Fig. 3).

### Validation of DHMD’s antiviral effect against cellular attachment of HCV particles using ELISA-based virus binding analysis and confocal microscopy

**Figure 6:**
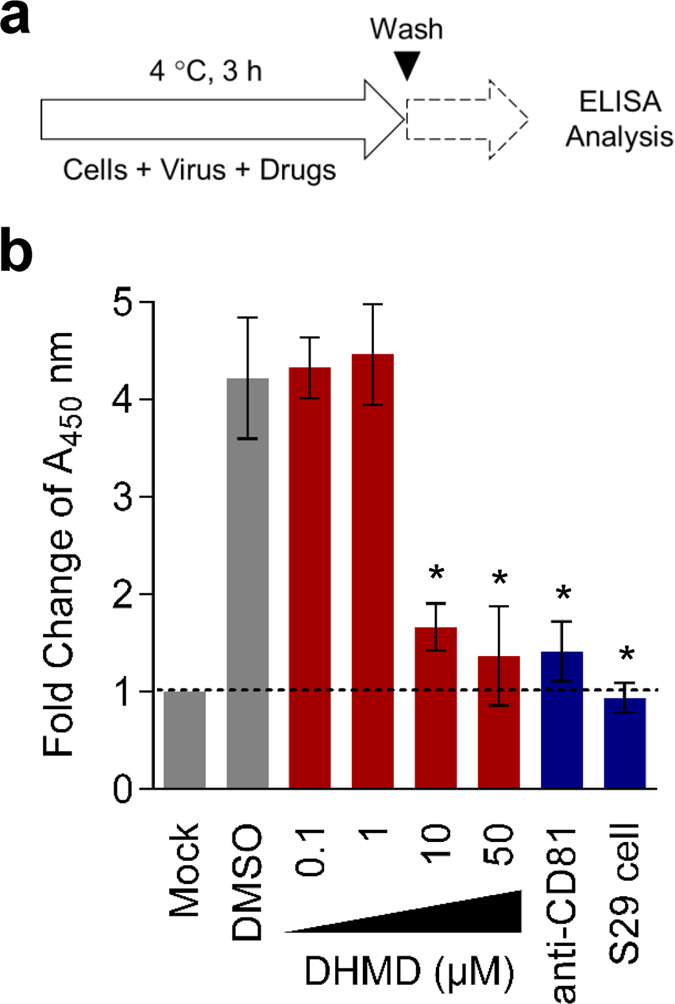
Confirmation of DHMD’s antiviral effect against HCV particle attachment using ELISA-based virus binding analysis. (**a**) Schematics of the ELISA-based virus binding assay. (**b**) Huh-7.5 cells were treated with DHMD (50 μM) and HCVcc (MOI = 0.01) at 4 °C for 3 h, then washed with PBS before fixation and analysis for cell surface-bound virus particles by ELISA using anti-HCV E2 antibody. Dashed line indicates baseline signals. DMSO = 0.5%; anti-CD81 = 10 μg/ml; S29 cell = CD81-deficient Huh-7 derivative. Data shown are means ± SEM (*P < 0.05) from 3 independent experiments.

Since DHMD’s antiviral effect appeared to be most prominent during the viral adsorption phase onto the host cell surface, we next used an enzyme-linked immunosorbent assay (ELISA)-based virus binding assay to validate this observation (Fig. 6a). DHMD was incubated with the HCVcc inoculum on the target Huh-7.5 cells at 4 °C before washing and fixing the cells for probing cell surface-bound virus by ELISA using antibody specific for the HCV E2 glycoprotein. Data indicated that treatment with DHMD effectively impeded HCV binding to the host cell surface in a dose-dependent manner (Fig. 6b). Similarly, treatment with anti-CD81 or the use of the CD81-deficient S29 cells (Huh-7 derivative) also prevented binding of HCVcc particles onto the cell surface. In contrast, treatment with the DMSO control had no impact in reducing cell-bound HCVcc (Fig. 6b).

**Figure 7:**
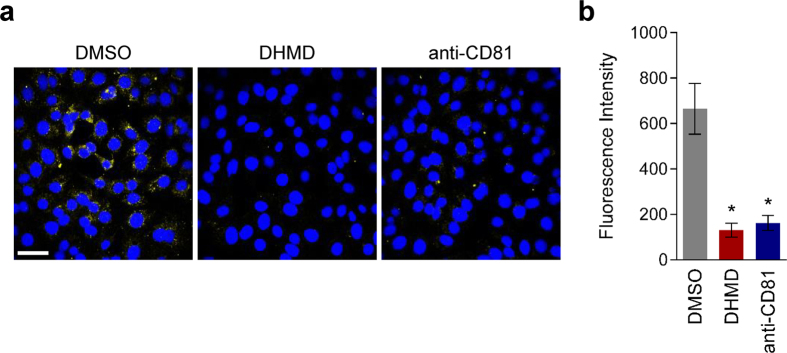
Confocal microscopy analysis of DHMD’s inhibitory effect against HCV adsorption. (**a**) Huh-7.5 cells seeded in chamber wells were co-incubated with HCVcc (MOI = 0.5) and DHMD (50 μM) or the controls at 4 °C for 3 h, then washed with PBS before shifting to 37 °C for 3 h. At the end of the incubation, cells were washed and fixed for immunostaining using anti-HCV core antibody and visualized with a confocal microscope. Nuclei were stained with the mounting medium used, which contained DAPI. DMSO = 0.5%; anti-CD81 = 10 μg/ml; magnification = 60×; scale bar = 50 μm. Data shown are means ± SEM (*P < 0.05) or representative micrographs from 3 independent experiments.

To further substantiate this result, we performed a viral entry analysis with confocal microscopy. HCVcc inoculum was co-incubated on Huh-7 cell surface with DHMD, DMSO, or anti-CD81 at 4 °C during the viral adsorption phase. The cells were then washed and shifted to 37 °C in fresh medium to allow completion of viral entry. After 3 h of incubation, the cells were immunostained for HCV core and analyzed with a confocal microscope to detect entry of viral particles that have successful attached to the host cell surface. As shown in Fig.7a,b, HCV core was readily detected in cells inoculated in the presence of DMSO, indicating that the solvent treatment had no impact on the viral adsorption step. On the contrary, very little core signals were detected in viral infections that were treated with DHMD or anti-CD81, suggesting that both treatments effectively blocked the earlier HCV particle binding onto the hepatoma cells. These observations therefore confirm and support the results obtained in Fig. 4c, whereby DHMD is highly efficacious at inhibiting viral attachment during early viral entry.

Altogether, the above studies identified DHMD as an efficient antagonist to HCV early viral entry, particularly targeting its viral adsorption step.

## Discussion

Several small molecules derived from natural products have been observed to inhibit HCV entry-related steps, including the green tea-derived catechin epigallocatechin-3-gallate^24^, the lectin griffithsin^25^, the flavonoid ladanein^26^, the hydrolyzable tannins chebulagic acid and punicalagin^27^, the diarylheptanoid curcumin^28^, the terpenoid saikosaponin b2^21^, and the trihydroxybenzoic acid gallic acid^29^. We report here for the first time that the butenolide DHMD isolated from *P. urinaria* is an efficient inhibitor to HCV early viral entry, specifically acting against viral attachment to the host cell surface. Our study therefore adds to the list of promising natural agents capable of antagonizing HCV entry, which could serve as potential candidates or leads for further development of HCV entry inhibitors.

Butenolides are lactones that are also regarded as furan derivatives. Several butenolides have been shown to exert antiviral activities, notably cochinolide against type I and II HSV infection^30^, 3-*epi*-litsenolide D_2_ against HIV replication^31^, and isoaspulvinone E against influenza virus neuraminidase activity^32^. In this study, we extracted three butenolides from *P. urinaria* including (−)-menisdaurilide (**1**), (4R,6R)-2-dihydromenisdaurilide (**2**), and (4R,6S)-2-dihydromenisdaurilide (**3**; DHMD). Both compounds **1** and **2** possess the same basal chemical skeleton, with their C-4 and C-6 in R form of steric configuration, while showing a difference of chemical bond between C-2 and C-3. Despite this difference, the anti-HCV activity of both compounds **1** and **2** are weak, indicating that the presence or absence of the double bond between C-2 and C-3 therefore does not appear to affect the bioactivity of either compounds. On the other hand, compounds **2** and **3** both possess a C-4 in R form while differing only in the chiral center of the C-6 in R and S form, respectively. Our experiment indicated that only compound **3** (DHMD) with a C-6 in S form displayed antiviral activity against HCV. Therefore, we speculate that the S form of the C-6 in DHMD plays a critical role in conferring its bioactivity against HCV (Fig. 2). Furthermore, a preliminary analysis indicated that DHMD does not exert antiviral activity against other tested viruses, including dengue virus type 2 (DENV-2), HSV-1, and measles virus (MV), suggesting that it may selectively inhibit HCV (Supplementary Fig. S4). Since DHMD exerted strong inhibitory activity against HCV particle binding (Figs 4 and 6) but had little influence in pretreatment of the host cell (Fig. 3), we hypothesize that DHMD likely targeted the HCV glycoproteins E1 and/or E2 to obstruct their receptor interactions or conformational changes rather than directly block the host cell surface receptors. This speculation is supported by the observation that DHMD could inactivate cell-free HCV particles and hence suggests an interaction between the compound and the virus (Fig. 4). Further in-depth analysis would be required to clarify the precise underlying mechanism.

Current DAAs all target the HCV non-structural proteins to inhibit its replication^2^,^4^. The development of inhibitors against other stages of the viral life cycle, including entry, can help expand the scope of antiviral strategies against hepatitis C. Specifically, HCV entry inhibitors would be useful as prophylactic treatment to prevent infection or re-infection (such as in liver transplant setting), and also as therapeutic treatment for restricting viral spread in infected individuals. In addition, as with the experience of HIV cocktail therapy, a combination treatment using multiple inhibitors that target the HCV life cycle is projected to maximize its treatment response rate^2^. Furthermore, inclusion of entry inhibitors to existing DAAs could impose a barrier to the drug resistance development, as more steps in the viral life cycle are being targeted. Inhibitors of HCV entry used in combination with DAAs has recently been shown to provide synergistic effects against HCV infection^33^, which further highlights the importance of developing such type of therapeutics. Our discovery of DHMD, which can efficiently inhibit HCV entry-related events, can therefore serve as a candidate agent or potential lead for the development of HCV entry inhibitors.

In conclusion, due to its potency in targeting HCV early viral entry, we suggest that DHMD may be of value for further development as a small molecule inhibitor for the treatment of hepatitis C, particularly for application in transplant setting.

## Materials and Methods

### Plant material and compound extraction

Fresh specimen of *Phyllanthus urinaria* (996.2 g) was extracted with acetone 3 times and subsequently fractionated by silica gel column chromatography using acetone/n-hexane and methanol/acetone mixtures to yield 18 subfractions. Fraction 15 (methanol:acetone = 1:5) was further processed with silica gel column chromatography to isolate compounds **1**–**3**, which are (−)-menisdaurilide (**1**), (4R,6R)-2-dihydromenisdaurilide (**2**), and (4R,6S)-2-dihydromenisdaurilide (**3**). The structures (Fig. 1) of these compounds were confirmed using nuclear magnetic resonance (NMR; ^1^H and ^13^C) (Supplementary Tables S1 and 2 and Figs S1–3) and comparison with previous literature^34^. For treatment analysis, the test compounds were solubilized in dimethyl sulfoxide (DMSO).

### Cell culture

Huh-7.5 cells (provided by Charles M. Rice of the Rockefeller University) were cultured in Dulbecco’s modified Eagle’s medium (DMEM) containing 10% fetal bovine serum (FBS), 50 μg/ml gentamicin, and 0.5 μg/ml amphotericin B (GIBCO-Invitrogen; Carlsbad, CA, USA).

### Virus production

Production of cell culture‐derived infectious HCV (HCVcc) tagged with Gaussia luciferase from the Jc1FLAG2(p7-nsGluc2A) construct (genotype 2a, provided by Dr. Charles M. Rice) has been described elsewhere^27^. HCV viral titer was expressed as focus forming units (FFU) and determined by immunofluorescence staining using primary anti-HCV NS5A mouse antibody (1:25,000; clone 9E10, gift from Dr. Charles M. Rice), followed by Alexa Fluor 488 goat anti-mouse immunoglobulin G (IgG) (H+L) (1:400; Invitrogen) as previously reported^27^.

### Cytotoxicity assay

Cytotoxicity analysis of the test compounds at various concentrations and determination of their respective CC_50_ values were performed on Huh-7.5 cells using the 2,3-bis-(2-methoxy-4-nitro-5-sulfophenyl)-2H-tetrazolium-5-carboxanilide (XTT) assay kit (Sigma; St. Louis, MO, USA) as previously described^22^.

### Viral inhibition assay

Huh-7.5 cell monolayers in 96-well plates (seeded at 1 × 10^4^ cells/well) were challenged with the HCVcc inoculum (100 FFU/well) for 3 h at 37 °C, in the presence of increasing concentrations of the test compounds, as a mixture prepared in DMEM containing 2% FBS. The cell monolayers were then washed with phosphate buffered saline (PBS) and further incubated at 37 °C for 72 h. After the 3-day incubation, cells were PBS-washed and fixed with ice-cold methanol before analyzing the viral infectivity by immunofluorescence staining of viral foci using the anti-NS5A 9E10 antibody, as mentioned above. The NS5A-positive viral foci were counted and data were plotted as percent (%) HCV inhibition against the DMSO (0.5%) negative control treatment. The EC_50_ value of each compound was determined using the GraphPad Prism 7 software (San Diego, CA, USA)^27^.

### Time-of-drug-addition assay

The time-of-drug-addition assay was performed using an earlier described method^22^, by adding DHMD (50 μM) to Huh-7.5 cell monolayers (1 × 10^4^ cells/well of a 96-well plate) either 24 h before, concurrently with, or subsequent to HCVcc inoculation (multiplicity of infection [MOI] = 0.01). Wash steps were included to ensure that the drug was only present during the specific treatment period (Fig. 3a). Following 72 h of incubation, the supernatant was collected and luciferase reporter signals reflecting viral infectivity was determined using the *Gaussia* luciferase assay kit (New England Biolabs; Pickering, ON, Canada) and a luminometer (Promega; Madison, WI, USA) as previously published^20^. Data are expressed as log_10_ of relative light units (log_10_ RLU). For comparison, IFN-α (1,000 international unit [IU]/ml; Sigma) and DMSO (0.5%) served as positive and negative control treatments, respectively.

### Synchronized infection analysis of early viral entry

The synchronized infection analysis of early viral entry including viral inactivation, viral attachment, and viral entry/fusion assays were performed as previously described^29^. A schematic for the specific procedure and incubation times is shown in Fig. 4a. Viral infectivity (log_10_ RLU) was detected by luciferase assay as described above.

### Viral replication/translation assay

Huh-7.5 cells were electroporated with HCV Jc1FLAG2 (p7-nsGluc2A) RNA (10 μg) and then seeded in culture plates. The next day, the cells were washed and treated with media containing the test compound. Accumulated reporter signals correlating with replication/translation of the viral genome were determined following 6 days of incubation and expressed as log_10_ RLU^21^.

### Viral assembly/release assay

Huh-7.5 cells were electroporated with the HCV Jc1FLAG2(p7-nsGluc2A) RNA genome (10 μg) and incubated for 24 h before refreshing with media containing the test compound. After 6 days of incubation, the cells and supernatants were harvested. The supernatants were further precipitated using polyethylene glycol-8000 (Sigma) and centrifuged to obtain pellet. Intracellular and extracellular RNAs were then extracted with Trizol (Invitrogen) from the harvested cells and supernatant-derived pellet, respectively. Quantitation of HCV genome from the obtained intracellular and extracellular RNAs by qRT-PCR was performed as previously described^21^.

### VSV plaque reduction assay

The plaque reduction assay using the IFN-sensitive VSV-EGFP^23^ for the infection of Huh-7.5 cells was performed following an earlier described method^22^. Huh-7.5 cells (5 × 10^5^/well) seeded in 12-well plates were treated with the test compound for 24 h. Cells were then washed with PBS twice and inoculated with VSV-EGFP (200 plaque forming units [PFU]/well). After 1 h of infection, the cells were washed again with PBS and overlaid with media containing 1% methylcellulose. Fluorescent viral plaques were subsequently scanned and quantitated at 24 h postinfection using the Typhoon 9410 variable mode imager (Amersham Biosciences; Baie d’Urfe, Quebec, Canada). Data were calculated as percent inhibition against VSV-EGFP infection relative to the DMSO treatment control.

### ELISA-based virus binding assay

Huh-7.5 cells were pre-chilled at 4 °C for 1 h and then treated with HCVcc (MOI = 0.01) in the presence of various concentrations of the test compound at 4 °C for an additional 3 h. Following virus adsorption, the cell monolayers were washed twice with ice-cold PBS before fixation with pre-chilled 4% paraformaldehyde (Sigma). Detection of cell surface-bound virus through ELISA was performed as previously reported^27^ using the anti-HCV E2 primary antibody (1: 20,000; Austral Biological, San Ramon, CA, USA), followed by a goat anti-mouse IgG conjugated with horseradish peroxidase secondary antibody (1: 36,000; Invitrogen), and developed with a TMB (3,3′,5,5′-tetramethylbenzidine) Two-component Microwell Peroxidase Substrate Kit (KPL; Gaithersburg, MD, USA). Results were measured with an ELx800 Microplate reader (Instrument, Inc.; Winooski, VT, USA) at 450 nm and data were expressed as fold change of A_450 _nm readings. Anti-CD81 (10 μg/ml; BD Biosciences, San Diego, CA, USA) treatment and the Huh-7-derived S29 cells, which lack CD81^35^, were included as positive controls, whereas DMSO (0.5%) treatment served as negative control. A schematic for the procedure is depicted in Fig. 6a.

### Confocal microscopy

DHMD (50 μM) and the HCV inoculum (MOI = 0.5) were concurrently incubated with Huh-7.5 cells seeded in Lab-Tek II chamber slides (1 × 10^5^ cells/well; Nalge Nunc International Corp., Penfield, NY, USA) at 4 °C for 3 h during the viral adsorption phase. The cells were then washed twice with ice-cold PBS before transferring to 37 °C for 3 h in complete medium to allow viral entry. Cells were subsequently washed again with PBS and fixed with ice-cold methanol. HCV positive cells were stained using mouse anti-core antibody (1:800; Thermo Fisher Scientific, Waltham, MA, USA) and Alexa Fluor 488 goat anti-mouse IgG (H+L) (1:400; Invitrogen). Nuclei were visualized by treatment with Vectashield Mounting Medium containing 4′,6-diamidino-2-phenylindole (DAPI; Vector Laboratories, Burlingame, CA, USA). All images were obtained using an Olympus FluoView FV1000 confocal laser scanning microscope (Olympus America; Center Valley, PA, USA) with a 60× objective, and total fluorescence intensity from each image was analyzed by the associated Olympus FV10-ASW software (Olympus America). Anti-CD81 (10 μg/ml; BD Biosciences) treatment served as positive control.

### Statistical analysis

Statistical analysis was performed by one-way ANOVA. Difference between data (compared to DMSO control treatment) were considered statistically significant when *P* < 0.05. Data were presented as means ± standard errors of the means (SEM) from three independent experiments (n = 3).

## Additional Information

**How to cite this article**: Chung, C.-Y. *et al*. (4R,6S)-2-Dihydromenisdaurilide is a Butenolide that Efficiently Inhibits Hepatitis C Virus Entry. *Sci. Rep.* **6**, 29969; doi: 10.1038/srep29969 (2016).

## Supplementary Material

Supplementary Information

## Figures and Tables

**Table 1:** Summary of the antiviral activities of DHMD against the HCV life cycle.

Pretreatment	Coaddition			Postinfection	
	Free Virus Particles	Attachment	Entry/Fusion	Replication/Translation	Assembly/Release
−	+	+	+	−	−
