# Supplementary material for: (4R,6S)-2-Dihydromenisdaurilide is a Butenolide that Efficiently Inhibits Hepatitis C Virus Entry
Source: Sci Rep. 2016 Jul 18;6:29969. doi: 10.1038/srep29969 (PMC4947960; doi:10.1038/srep29969)
Supplement: Supplementary Information [file srep29969-s1.pdf]

SUPPLEMENTARY INFORMATION

(4R,6S)-2-Dihydromenisdaurilide is a Butenolide that Efficiently Inhibits Hepatitis C Virus Entry

Chueh-Yao Chung[a], Ching-Hsuan Liu[b], Guey-Horng Wang[c], Alagie Jassey[b,d], Chia-Lin Li[b], Lei Chen[b], Ming-Hong Yen[a], Chun-Ching Lin[a,e,**], and Liang-Tzung Lin[b,d,*]

[a] Graduate Institute of Natural Products, College of Pharmacy, Kaohsiung Medical University, Kaohsiung, Taiwan
[b] Department of Microbiology and Immunology, School of Medicine, College of Medicine, Taipei Medical University, Taipei, Taiwan
[c] Research Center of Natural Cosmeceuticals Engineering, Xiamen Medical College, Xiamen City, China
[d] Graduate Institute of Medical Sciences, College of Medicine, Taipei Medical University, Taipei, Taiwan
[e] School of Pharmacy, College of Pharmacy, Kaohsiung Medical University, Kaohsiung, Taiwan

Supplementary Table S1. ^{13}C spectral data of compounds **1-3**.

	(-)-Menisdaurilide (Compound **1**)		(4R,6R)-2-Dihydromenisdaurilide (Compound **2**)		(4R,6S)-2-Dihydromenisdaurilide (Compound **3**)	
1	162.6	166.3‡	169.9	174.0	173.1	175.4
2	120.1	120.4	24.2	25.0	23.2	23.8
3	143.1	146.1	35.8	35.8	33.9	34.1
4	66.9	67.3	67.0	67.4	64.9	66.4
5	40.0	41.1	42.2	43.3	41.5	41.6
6	77.9	80.1	79.7	81.6	79.6	80.9
7	111.7	111.5	113.5	113.4	112.3	112.6
8	173.1	176.0	173.2	176.0	173.7	176.3

‡Red color indicates literature spectral data[34].

Supplementary Table S2. ^{1}H spectral data of compounds **1-3**.

	(-)-Menisdaurilide (Compound **1**)		(4R,6R)-2-Dihydromenisdaurilide (Compound **2**)		(4R,6S)-2-Dihydromenisdaurilide (Compound **3**)	
2	6.58	6.63‡	2.35<br>2.88	2.41<br>2.87	2.65<br>2.85	2.76<br>2.76
3	6.32	6.43	1.44<br>2.26	1.36<br>2.18	1.43<br>2.09	1.58<br>2.09
4	4.65	4.58	3.94	3.89	4.35	4.25
5	1.65<br>2.96	1.69<br>2.96	1.38<br>2.77	1.24<br>2.68	1.37<br>2.76	1.40<br>2.64
6	4.88	4.94	4.75	4.91	5.37	5.13
7	5.84	5.86	5.75	5.80	5.83	5.78

‡Red color indicates literature spectral data[34].

Supplementary Figure S1.

^{13}C spectral data of (-)-menisdaurilide (compound **1**)

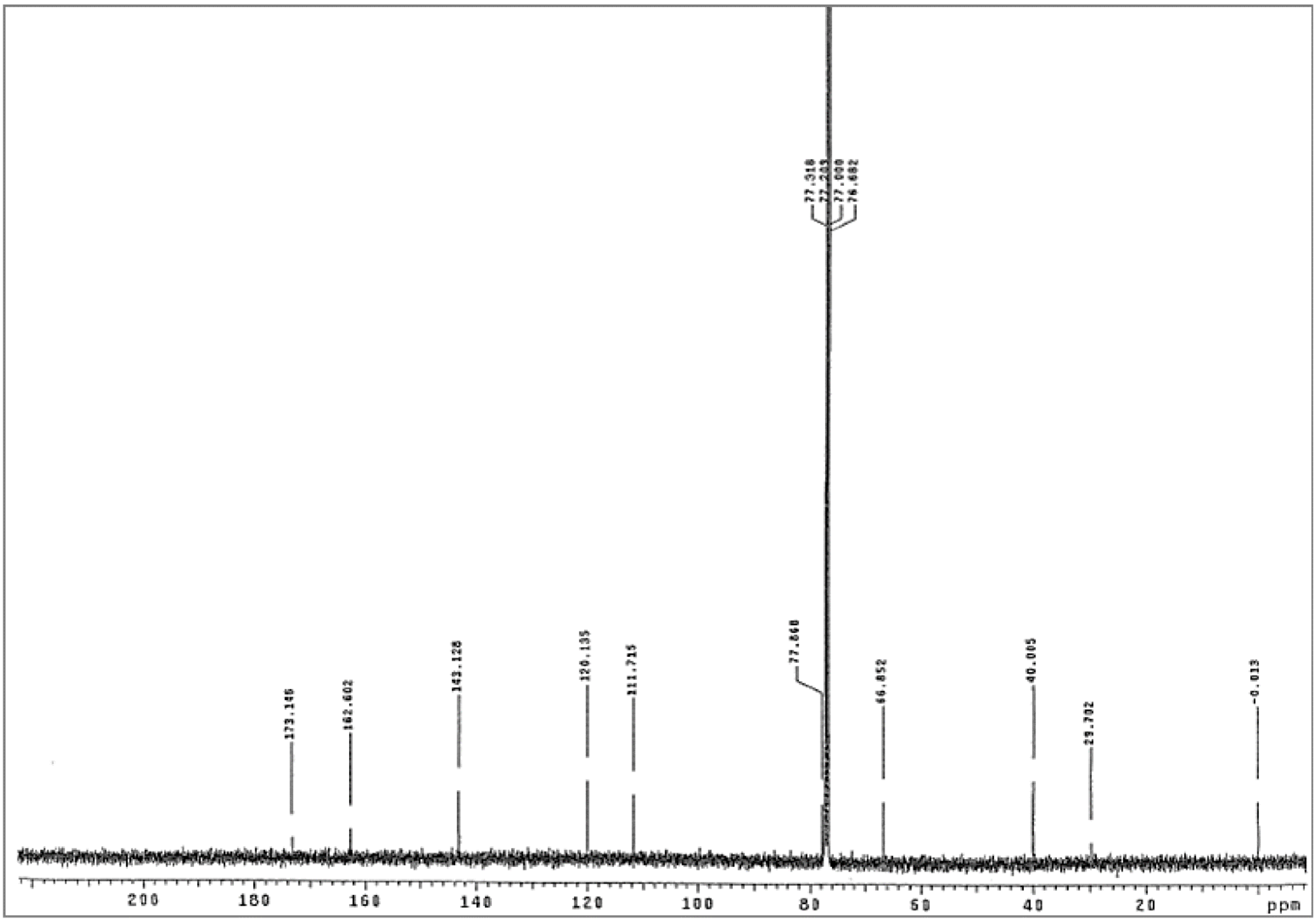

^{1}H spectral data of (-)-menisdaurilide (compound **1**)

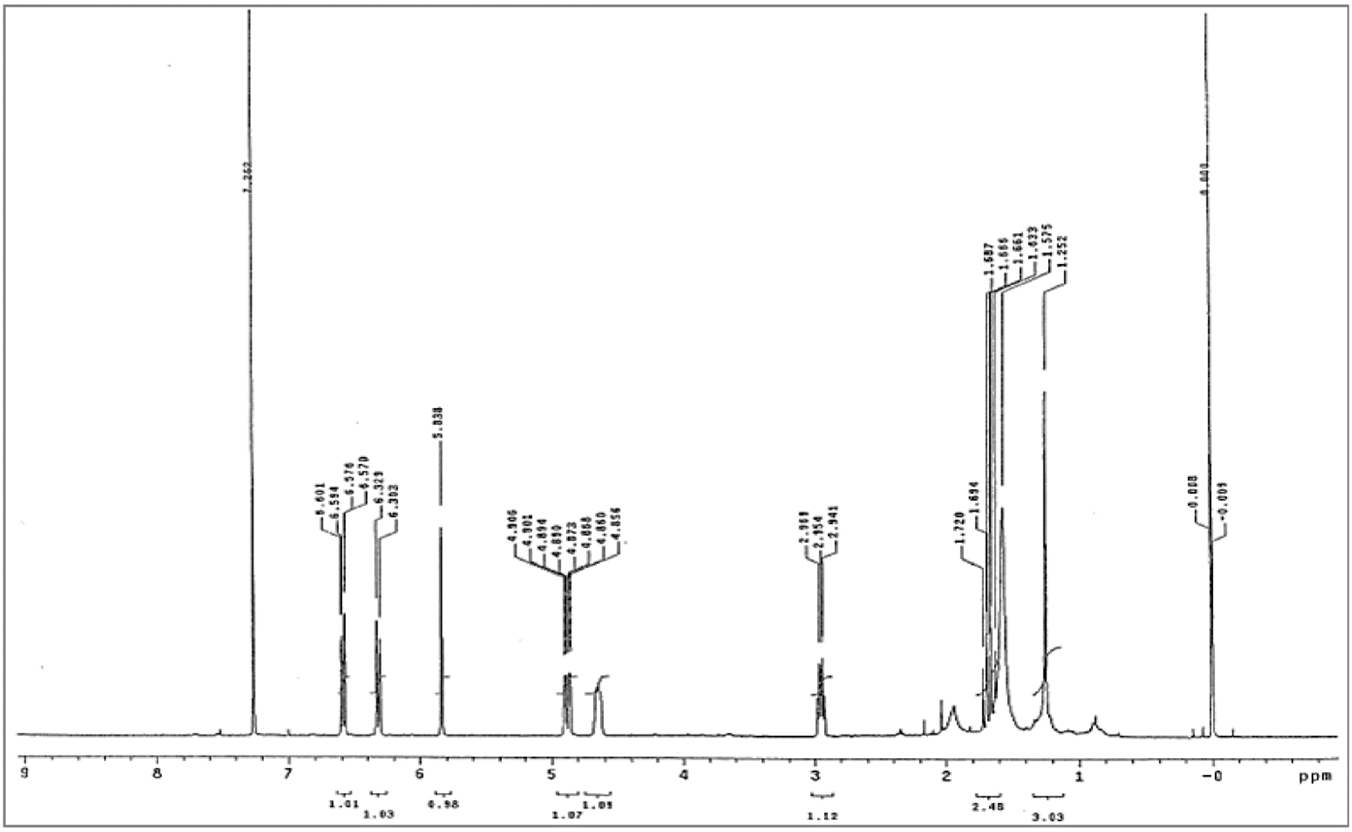

Supplementary Figure S2.

^{13}C spectral data of (4R,6R)-2-dihydromenisdaurilide (compound **2**)

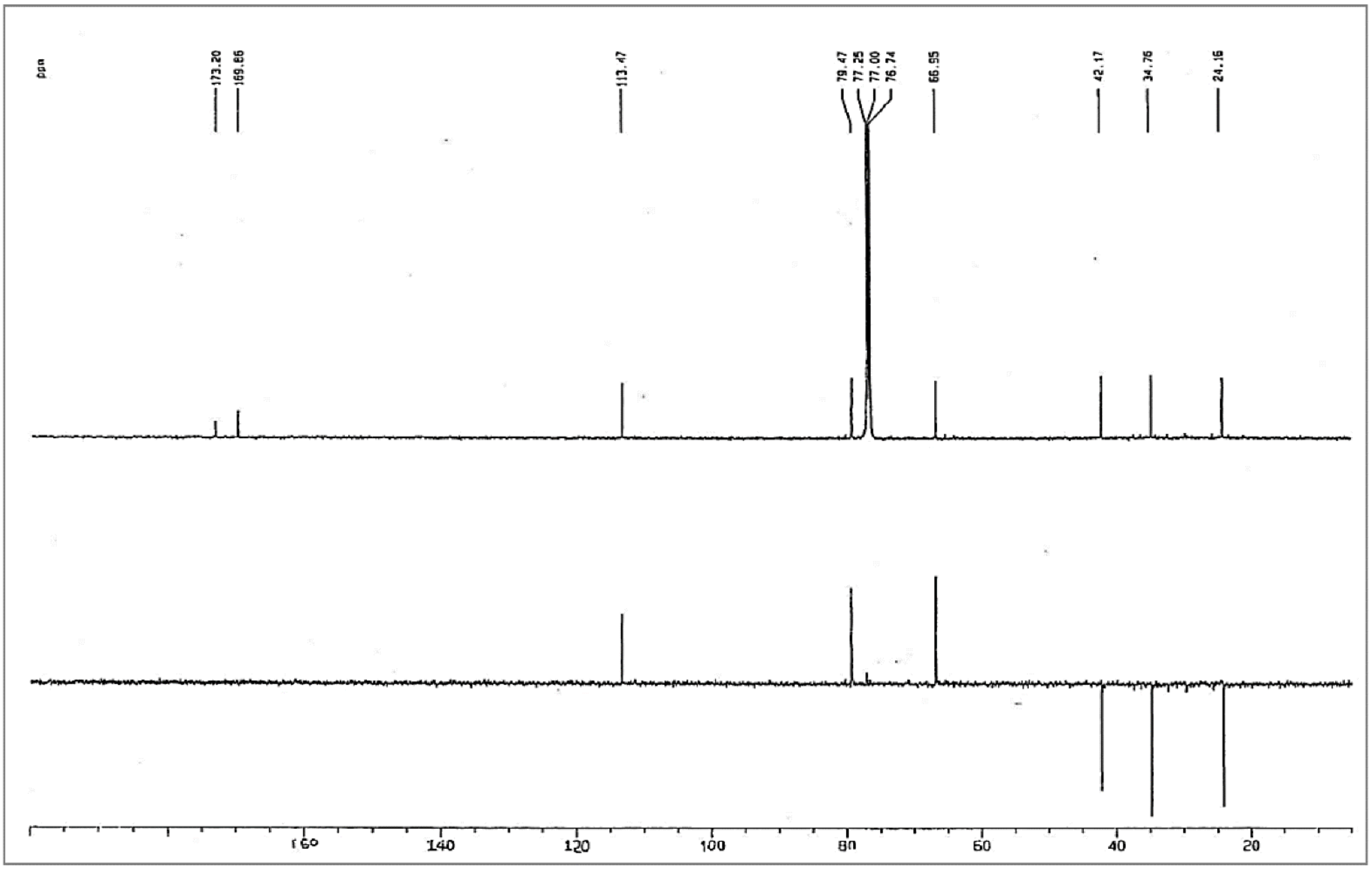

^{1}H spectral data of (4R,6R)-2-dihydromenisdaurilide (compound **2**)

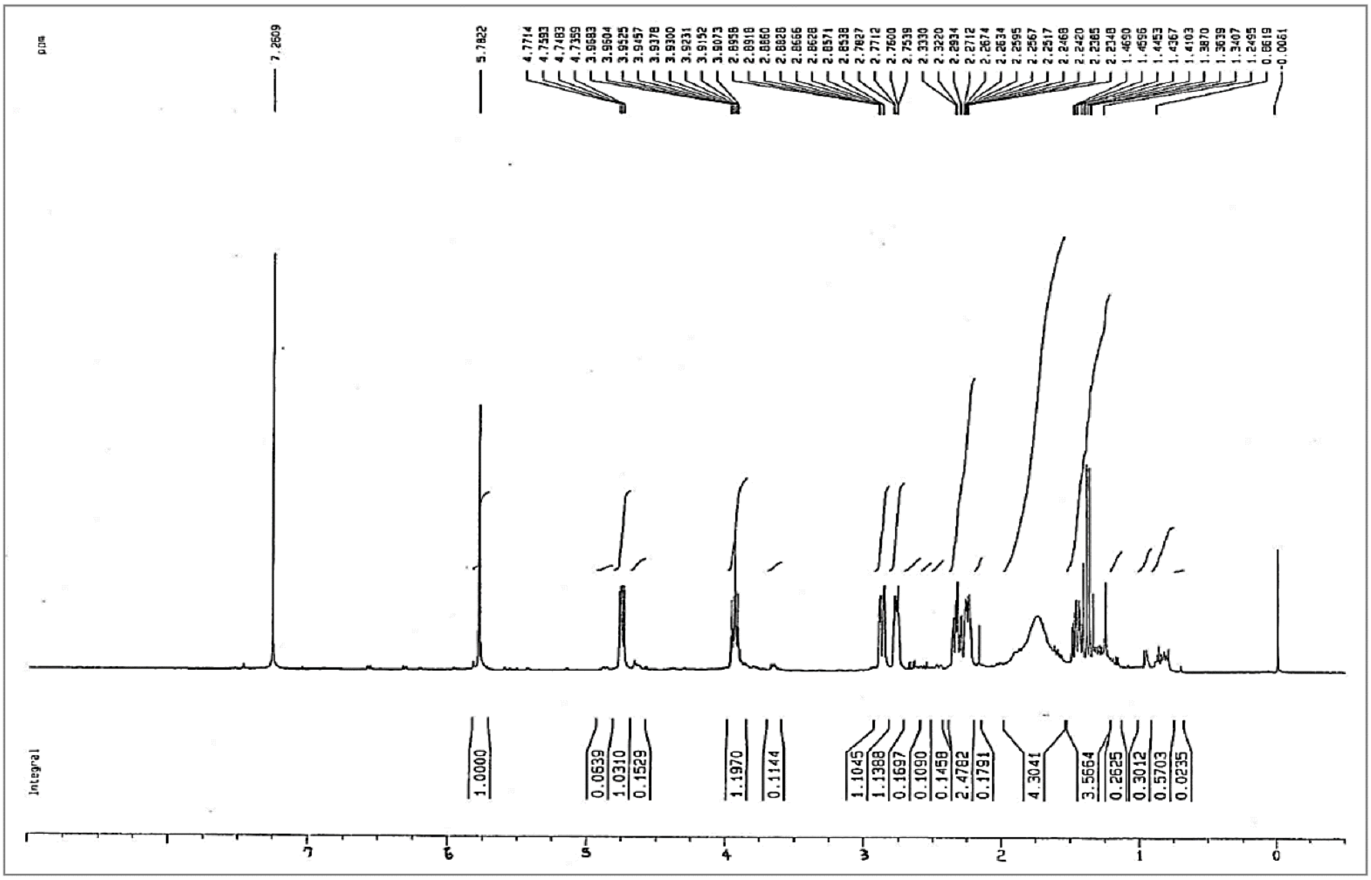

Supplementary Figure S3.

^{13}C spectral data of (4R,6S)-2-dihydromenisdaurilide (compound **3**)

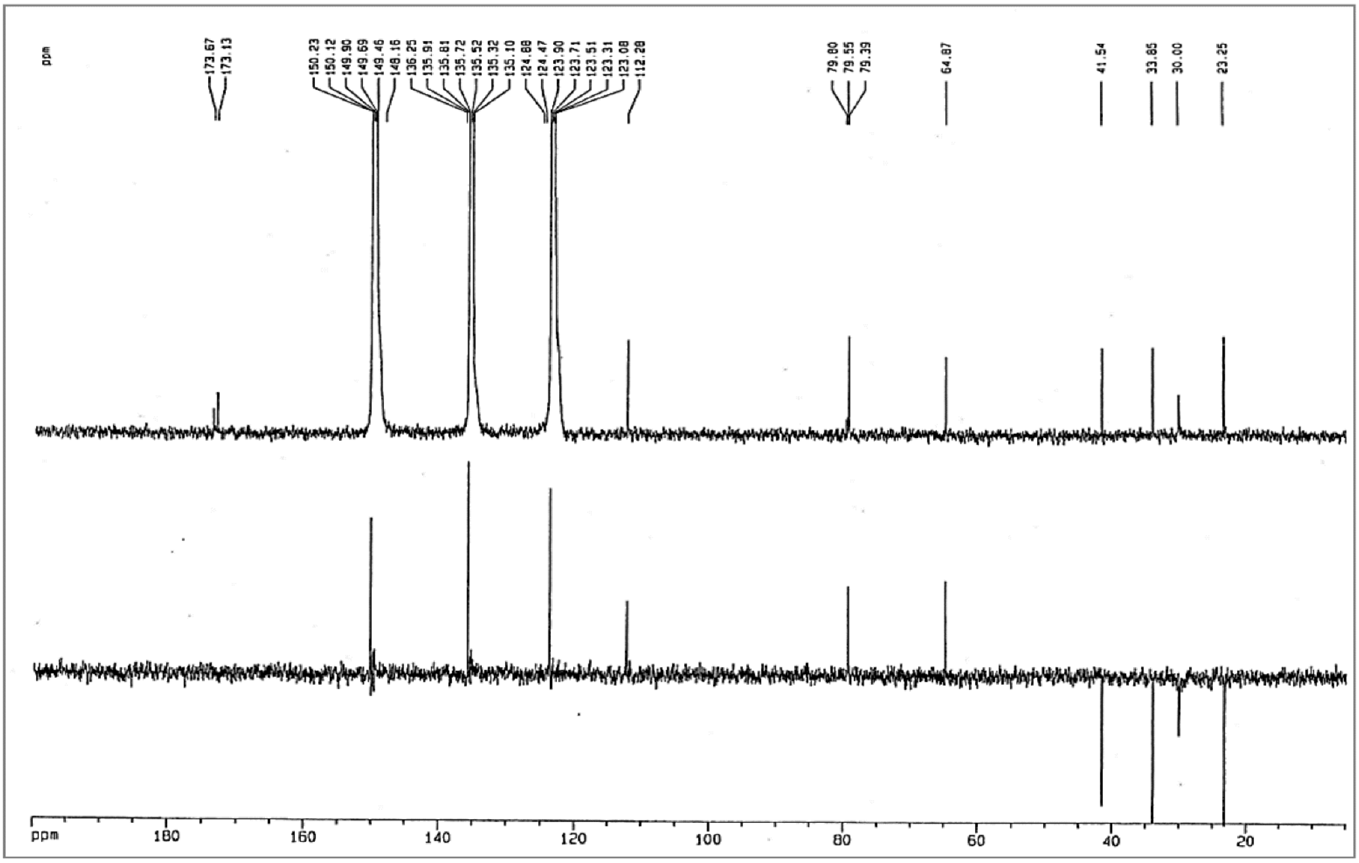

^{1}H spectral data of (4R,6S)-2-dihydromenisdaurilide (compound **3**)

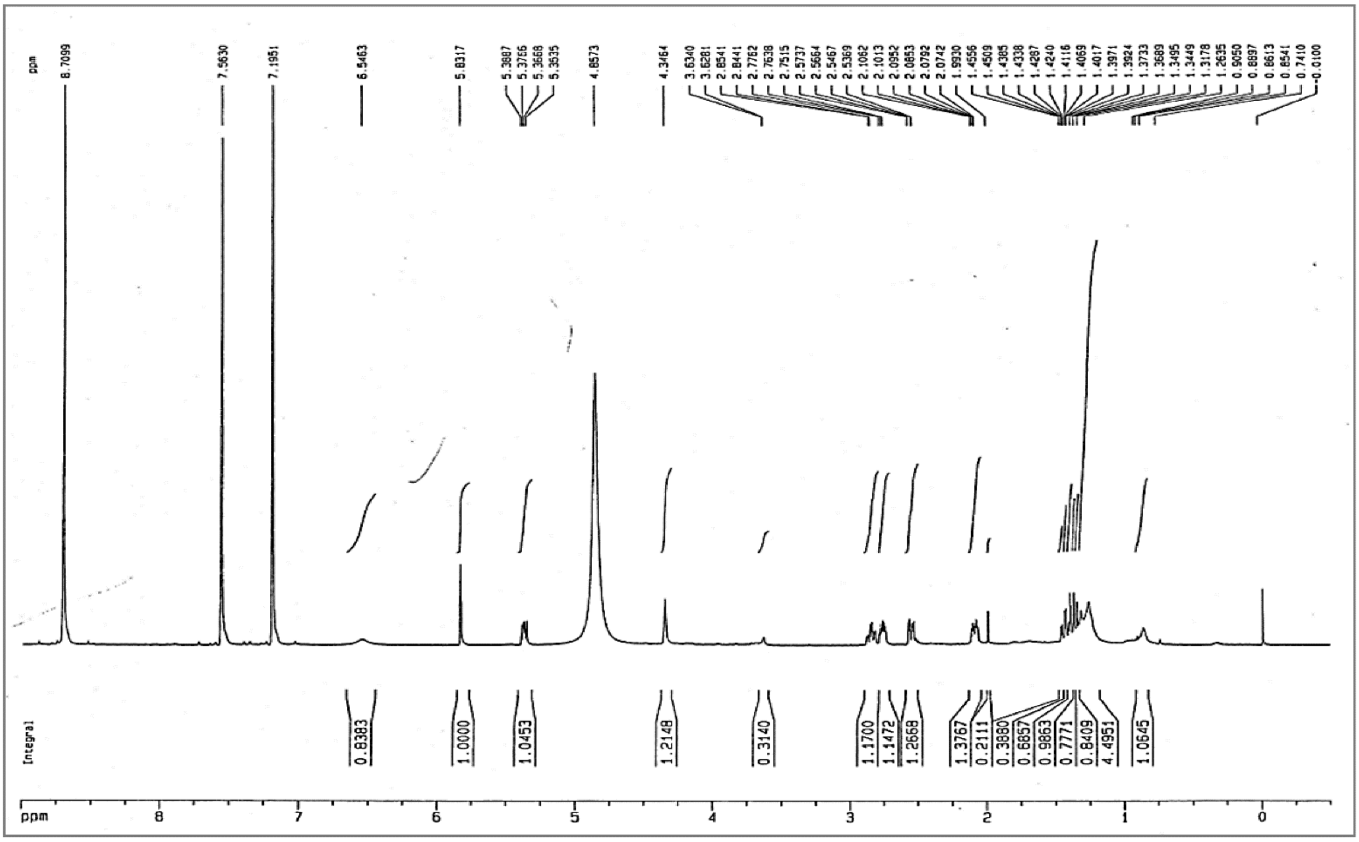

Supplementary Figure S4.

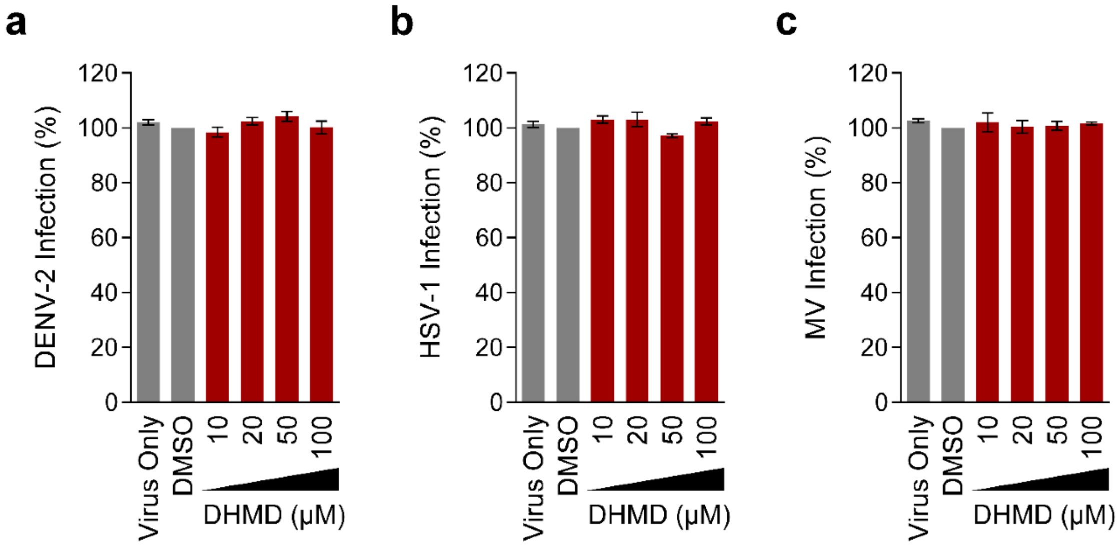

Supplementary Fig. S4. Effect of DHMD treatment on other viral infections. (a) Vero cells were cultured in 12-well plates (3 × 10^5 cells/well) overnight and infected with DENV-2 (100 PFU/well) for 1.5 h before addition of test compounds in overlay media containing 0.75 % methylcellulose. After 6 days of incubation, cells were fixed and analyzed by immunohistochemical staining-based plaque assay using anti-flavivirus group antibody (1:1,000; Millipore, Billerica, MA, USA) and a goat anti-mouse IgG (H + L) alkaline phosphatase (AP)-conjugated secondary antibody (1:5,000; Invitrogen). (b) A549 cells were cultured in 12-well plates (3 × 10^5 cells/well) overnight and infected with EGFP-tagged HSV-1 (200 PFU/well) for 1 h before addition of test compounds in overlay media containing 1 % methylcellulose. After 2 days of incubation, fluorescent viral plaques were quantitated using a Typhoon 9410 variable mode imager (Amersham Biosciences; Baie d'Urfe, Quebec, Canada). (c) CHO-SLAM cells were cultured in 96-well plates (1 × 10^4 cells/well) overnight and infected with EGFP-tagged MV (MOI = 0.03) for 1 h before addition of test compounds. After 2 days of incubation, fluorescent viral plaques were quantitated with a Typhoon 9410 variable mode imager. All results are expressed as percent (%) viral infection against DMSO (0.5 %) control treatment. Data shown are mean ± SEM from 3 independent biological replicates.
